# Seed Priming with Gibberellic Acid Enhances Drought Tolerance in Sweet Sorghum by Modulating Cuticular Wax and Cutin Composition

**DOI:** 10.3390/plants15152325

**Published:** 2026-07-29

**Authors:** Sennan Li, Xia Lan, Luhua Yao

**Affiliations:** Department of Agriculture and Forestry, Hainan Tropical Ocean University, Sanya 572022, China; 17733100454@163.com (S.L.); lanxia1108@163.com (X.L.)

**Keywords:** cuticular wax, cutin, GA priming, sweet sorghum

## Abstract

Drought stress severely constrains sorghum productivity, and seed priming has emerged as a potential strategy to enhance stress resilience. However, the role of cuticle deposition in seed priming-mediated drought tolerance remains unclear. In this study, the optimal GA priming concentration was determined and its effects on cuticular wax and cutin composition, as well as leaf water loss, were investigated in sorghum seedlings. A preliminary experiment identified 10 mg L^−1^ GA as the optimal dose, as it produced the greatest height (11.5%) and biomass (79.8%) under drought stress while significantly reducing MDA and O_2_^−^ levels, indicating effective alleviation of oxidative damage. Under well-watered conditions, GA priming moderately increased alkanes (45.6%) and primary alcohols (16.3%) while reducing aldehydes (27.3%). However, under drought conditions, GA induced substantially greater increases in alkanes (76.4%), primary alcohols (257.3%), amyrin (451.5%), and other alcohols (56.3%). Regarding cutin monomers, GA further elevated alkanoic acids and cyclopropaneoctanoic acid by 58.2% and 31.4%, respectively, on top of drought-induced accumulation, indicating a synergistic effect between GA and drought signals in promoting cuticular deposition. Additionally, GA redirected the production of cutin with a chain length of C16/C18 towardC22 cutin. GA-primed plants displayed the lowest water loss rates, correlating with enhanced cuticular deposition. Collectively, these findings demonstrate that, in the single genotype tested under controlled-environment conditions, GA priming enhances drought tolerance in sorghum seedlings, acting through the modulation of cuticle composition to reduce leaf water loss while alleviating oxidative damage and improving growth.

## 1. Introduction

Drought stress constitutes a primary constraint on global crop productivity, and its frequency and severity have increased concomitantly with climate change [1]. Developing strategies to improve drought tolerance has therefore become a key research priority. Seed priming is a pre-sowing hydration treatment that activates preparatory metabolism and repair, thereby improving seedling vigor and subsequent drought tolerance [2,3]. Among various priming agents, gibberellins (GAs) have attracted considerable interest due to their dual functions in promoting growth and modulating stress responses [4].

Seed priming with GAs has been reported to alleviate drought-induced inhibition of germination and seedling growth in multiple crop species. In soybeans, GA3 priming promotes germination and seedling growth under drought conditions by enhancing osmotic adjustment and antioxidative defenses [5]. In tomato, nano-encapsulated GA priming promotes plant growth under well-watered conditions and alleviates leaf dehydration under water deficit while improving water use efficiency [6]. Similarly, GA priming has been shown to enhance drought tolerance in rapeseed [7] and alfalfa [8], as reflected by improved seedling growth and germination performance. Although these studies demonstrated consistent positive effects of GA seed priming on drought tolerance across species, whether this improvement involves regulation of the cuticle—the primary barrier against water loss—remains unexplored.

The plant cuticle is a hydrophobic lipid layer on epidermal cell walls that restricts non-stomatal water loss [9,10]. Chemically, it comprises two complementary components: cutin and cuticular waxes. Cutin is a polyester network of hydroxylated and epoxy-hydroxy fatty acids that provides mechanical strength [11,12]. Cuticular waxes, composed of very-long-chain fatty acids and their derivatives, govern leaf surface hydrophobicity and water permeability [13]. Extensive evidence indicates that water deficit triggers compensatory cuticle thickening in plants. In Arabidopsis and maize, drought stress significantly increases total wax coverage, accompanied by a shift towards longer-chain alkanes, a compositional adjustment that is believed to reduce cuticular permeability [14,15,16]. Concurrently, drought has been shown to stimulate cutin monomer accumulation, synergistically reinforcing cuticular water retention [14,17]. Thus, coordinated deposition of waxes and cutin is a key strategy for drought adaptation, and the coupling of these two components in structure and function ultimately determines the overall barrier function efficacy of the cuticle [18].

The effects of seed priming extend beyond germination to later developmental stages. This transmission occurs via a priming memory mechanism that modulates subsequent stress responses [19,20,21]. GA priming, in particular, accelerates germination, promotes radicle elongation, and regulates osmolyte accumulation [22,23]. Notably, previous work has demonstrated that seed priming with other hormones, such as abscisic acid (ABA), can significantly alter cuticular wax composition in sorghum seedlings [24], suggesting that hormone priming can modulate cuticle biosynthesis. While ABA signaling is well-established as a central regulator of drought tolerance [24], the present study focused specifically on the cuticular barrier as a downstream target, given that cuticle deposition directly determines leaf water retention. However, whether GA priming exerts similar effects on cuticular deposition remains unknown. Accordingly, it is hypothesized that GA seed priming enhances seedling drought tolerance by promoting cuticular component accumulation.

Sorghum (*Sorghum bicolor*), a C4 crop with inherent drought tolerance, holds significant importance in global food and bioenergy production. Its leaf surface has a dense cuticular coating [24,25]. Sweet sorghum, a variety characterized by high biomass yields and favorable stress tolerance, is a suitable system for investigating the relationship between cuticular traits and drought resistance.

In this study, we systematically compared seedling growth performance, leaf water loss rate, and cuticular chemical profiles (including both wax and cutin contents and monomer compositions) between GA-primed and non-primed sweet sorghum plants under well-watered and drought-stressed conditions. The findings of this study are expected to provide direct experimental evidence for the biochemical basis of GA priming-induced drought tolerance and to offer a theoretical foundation for the application of hormone-based seed priming in drought-prone agricultural systems.

## 2. Results

### 2.1. Effects of Seed Priming with Different Gibberellin Concentrations on Growth and Drought Tolerance of Sorghum Seedlings

**Figure 1:**
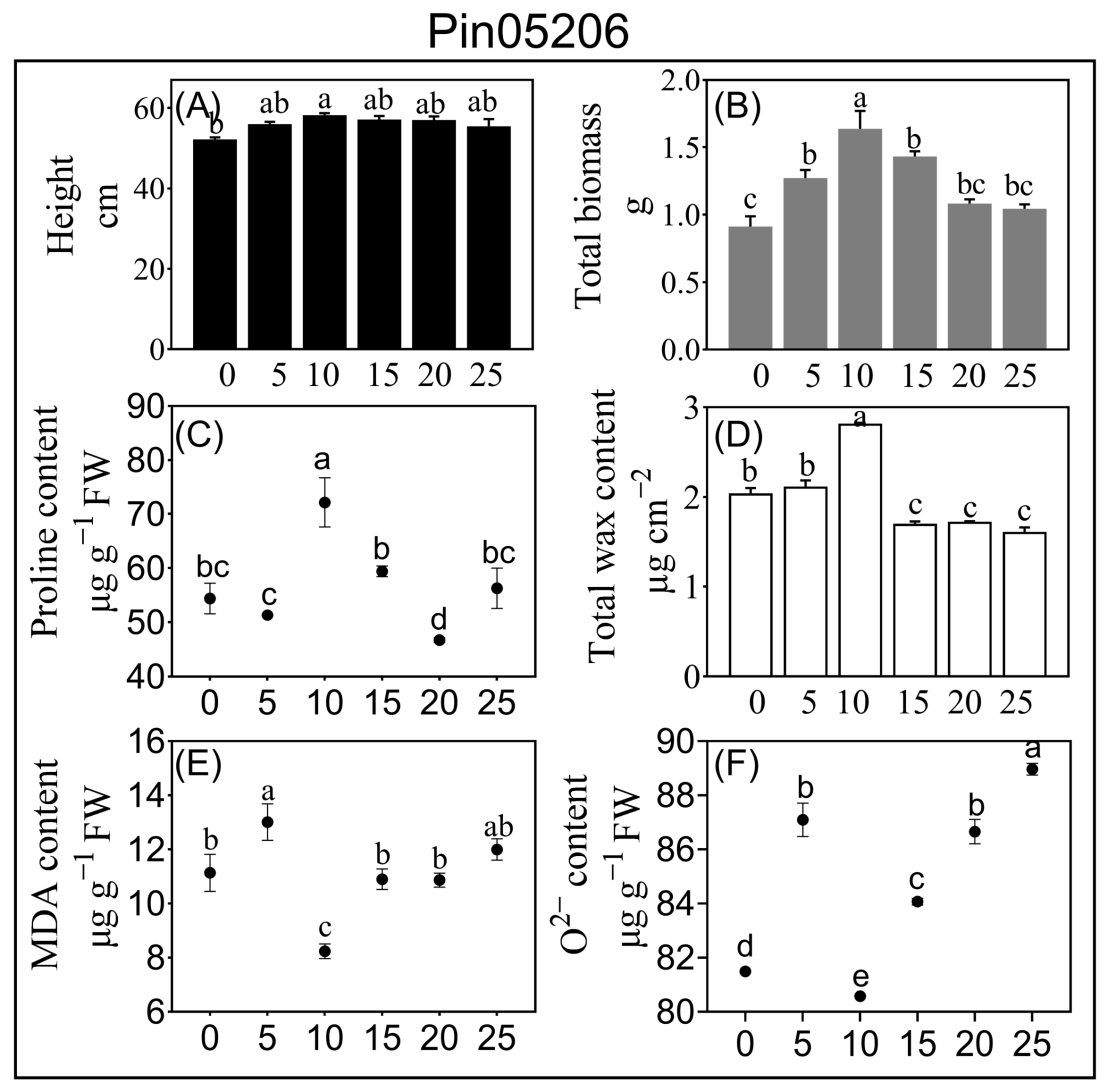
Effects of GA seed priming on sorghum growth and oxidative stress response: (**A**) plant height; (**B**) biomass; and (**C**) proline, (**D**) total wax, (**E**) malondialdehyde (MDA), and (**F**) superoxide anion (O^2−^) contents. The data are presented as the mean ± SE of three replicates. Different letters indicate significant differences between bars according to Tukey’s post hoc test (*p* < 0.05); bars sharing the same letter or without letters are not significantly different.

In a preliminary experiment, sweet sorghum cv. Pin05206, Hunnigreen and Mule8000 seeds primed with different GA concentrations were subjected to drought stress at the seedling stage (Figure 1 and Figures S1 and S2). Under drought conditions, as the GA priming concentration increased, plant height and biomass initially increased and then decreased in three varieties. In cv. Pin05206, the highest height and biomass were observed with the 10 mg L^−1^ treatment, which increased by 11.5% and 79.8%, respectively, compared to the drought-only treatment in cv. Pin05206. Additionally, the 10 mg L^−1^ GA-primed plants showed increased proline (32.7%) and total leaf wax contents (38.1%) compared to non-primed plants when subjected to drought stress (Figure 1C,D). The levels of MDA and O^2−^ decreased significantly in GA-primed plants compared to drought-treated plants (Figure 1E,F). Based on these screening results, 10 mg L^−1^ GA was selected as the optimal dose for the main experiment described in the following sections. Similar trends across all measured parameters were also observed for Hunnigreen and Mule8000, with 10 mg L^−1^ GA identified as the optimal concentration (Figures S1 and S2).

### 2.2. Effects of Seed Priming with Gibberellin on Cuticle Deposition

To formally evaluate the statistical significance of the observed effects, a two-way analysis of variance (ANOVA) was performed with water regime (well-watered vs. drought) and priming treatment (control, water-primed, GA-primed) as fixed factors, followed by Tukey’s HSD post hoc test for multiple comparisons (Table 1). The analysis revealed significant main effects of water regime and priming treatment, as well as significant water × priming interactions, for total wax (W: F_1,12_ = 43.45, *p* < 0.001; P: F_2,12_ = 92.31, *p* < 0.001; W × P: F_2,12_ = 45.41, *p* < 0.001) and total cutin (W: F_1,12_ = 193.89, *p* < 0.001; P: F_2,12_ = 163.06, *p* < 0.001; W × P: F_2,12_ = 56.09, *p* < 0.001). These results confirm that the effect of GA priming on cuticle deposition is dependent on water availability, with the priming-induced enhancement being substantially amplified under drought stress.

**Figure 2:**
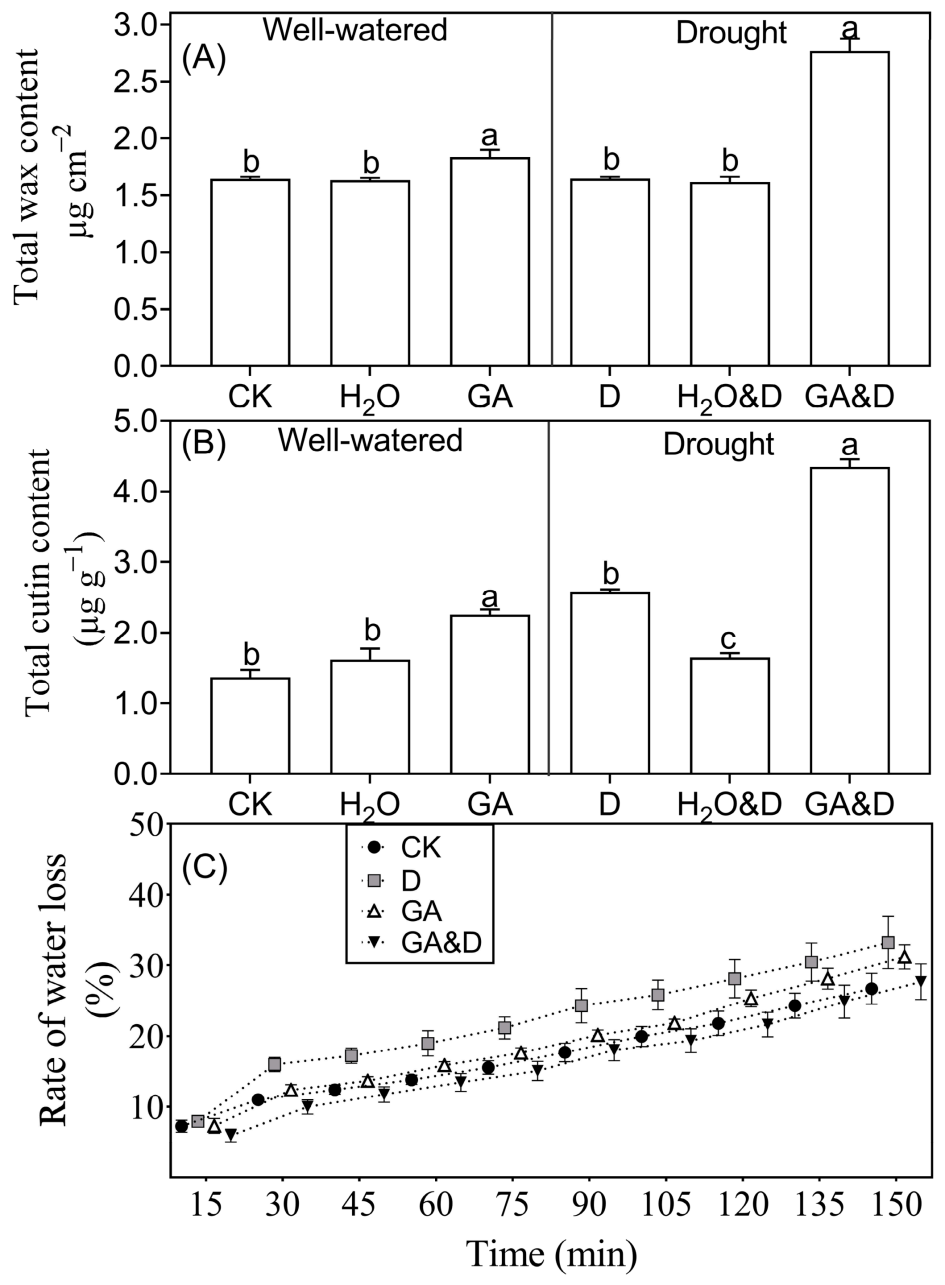
Effects of seed priming with GA on leaf total wax (**A**), cutin (**B**), and water loss rate (**C**). The data are presented as the mean ± SE (*n* = 3). CK, plants from non-primed seeds grown under well-watered conditions; D, plants from non-primed seeds grown under drought stress; H_2_O and GA, plants from H_2_O- and GA-primed seeds grown under well-watered conditions; H_2_O&D and GA&D, plants from H_2_O- and GA-primed seeds grown under drought stress. Different letters indicate significant differences between bars according to Tukey’s post hoc test (*p* < 0.05); bars sharing the same letter or without letters are not significantly different.

To further elucidate the influence of GA priming on cuticular deposition in sorghum, total leaf cutin and wax contents were analyzed (Figure 2). Under drought stress, GA-primed plants exhibited significantly higher total cutin and total wax contents than their non-primed counterparts, with increases of 68.9% and 68.1%, respectively (Figure 2A,B). Notably, this enhancing effect was also observed under well-watered conditions, where GA priming increased total wax by 11.7% and total cutin by 64.6% (Figure 2A,B). Drought stress alone significantly elevated the total cutin content but did not have a detectable effect on total wax accumulation relative to the well-watered control. To exclude any solvent-related artifacts, a water-priming control was included; this treatment showed no significant influence on total wax content, with the exception of a reduction in total cutin under drought stress (Figure 2A,B). Cuticular water loss measurements revealed that GA-primed plants maintained the lowest dehydration rate among all treatments (Figure 2C). Collectively, these findings indicate that GA priming alleviates drought-induced stress in sorghum, primarily through enhanced cuticular component deposition and reduced foliar water loss.

### 2.3. Effects of Seed Priming with Gibberellin on Cutin Monomer and Wax Components

**Figure 3:**
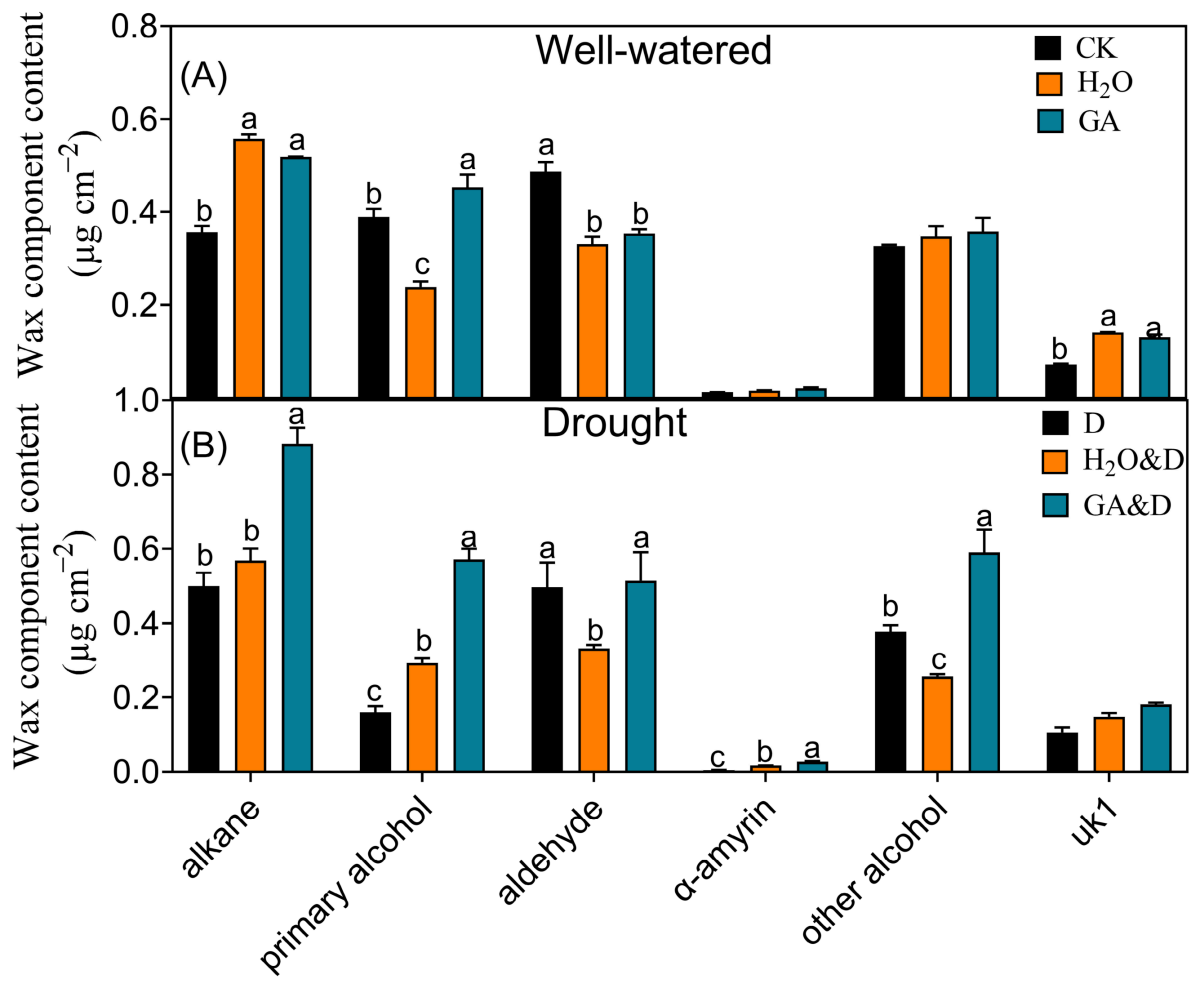
Effects of seed priming with GA on cuticular wax composition under (**A**) well-watered and (**B**) drought stress conditions. The data are presented as the mean ± SE (*n* = 3). uk1, unknown wax composition. CK, plants from non-primed seeds grown under well-watered conditions; D, plants from non-primed seeds grown under drought stress; H_2_O and GA, plants from H_2_O- and GA-primed seeds grown under well-watered conditions; H_2_O&D and GA&D, plants from H_2_O- and GA-primed seeds grown under drought stress. Different letters indicate significant differences between bars according to Tukey’s post hoc test (*p* < 0.05); bars sharing the same letter or without letters are not significantly different.

**Figure 4:**
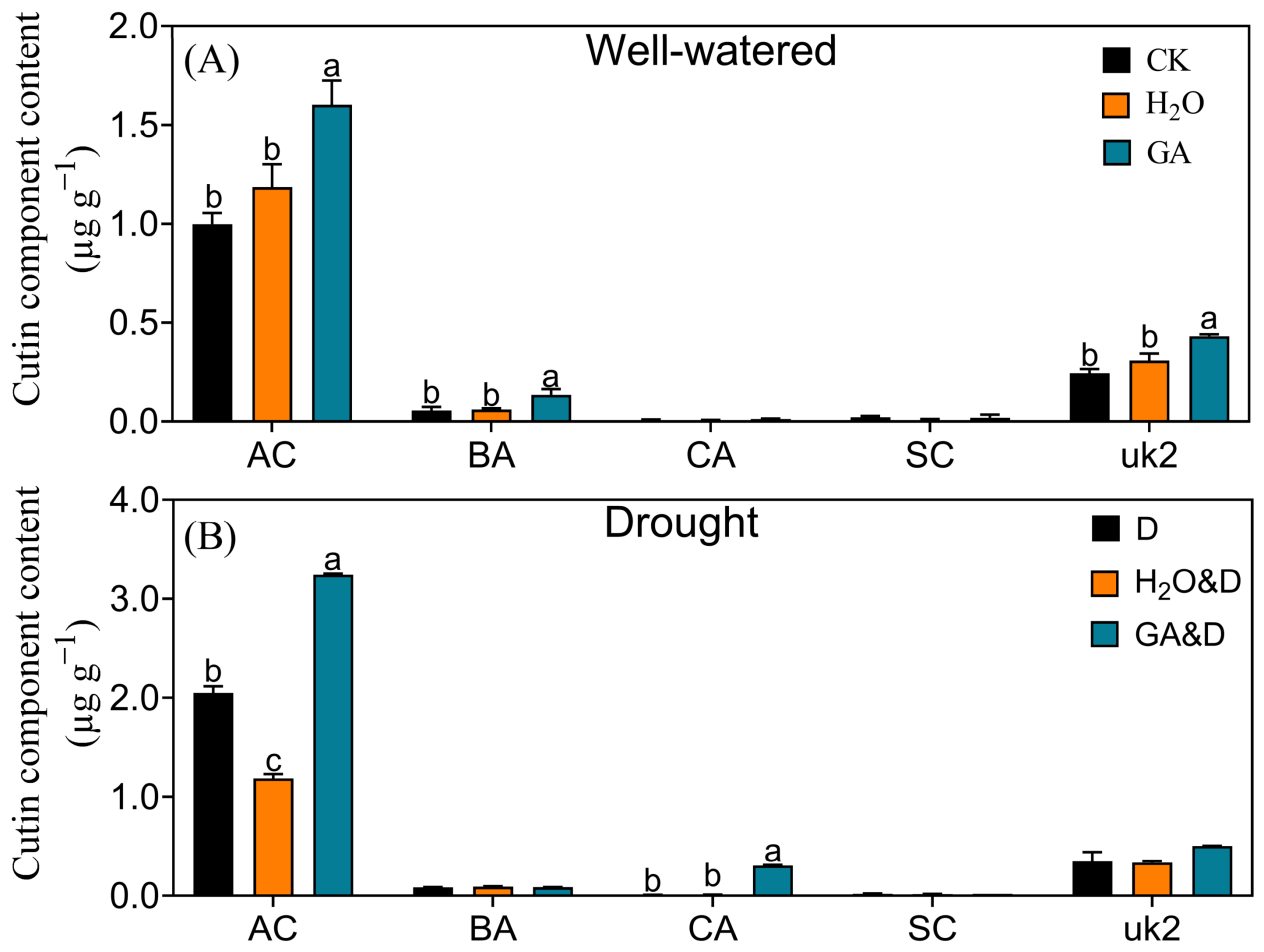
Effects of seed priming with GA on cutin monomer under (**A**) well-watered and (**B**) drought stress conditions. The data are presented as the mean ± SE (*n* = 3). uk2, unknown cutin monomers; AC, alkanoic acid; BA, branched alkanoic acid; CA, cyclopropaneoctanoic acid; SC, stearic acidd. CK, plants from non-primed seeds grown under well-watered conditions; D, plants from non-primed seeds grown under drought stress; H_2_O and GA, plants from H_2_O- and GA-primed seeds grown under well-watered conditions; H_2_O&D and GA&D, plants from H_2_O- and GA-primed seeds grown under drought stress. Different letters indicate significant differences between bars according to Tukey’s post hoc test (*p* < 0.05); bars sharing the same letter or without letters are not significantly different.

To further dissect the effects of GA priming on cuticle deposition, cutin monomers and wax constituents in sorghum leaves were quantified (Figure 3 and Figure 4). Sorghum leaf cuticular waxes consist of alkanes, alcohols (primary alcohols, simiarenol, and fernenol), aldehydes, and α-amyrin (a triterpenoid). GA priming elicited substantial changes in wax components under both well-watered and drought stress conditions (Figure 3). Under well-watered conditions, GA priming significantly increased the alkane content by 45.6% and primary alcohol content by 16.3%, while concurrently reducing the aldehyde content by 27.3% (Figure 3A). Under drought stress, GA priming induced more pronounced and broader changes: alkanes, primary alcohols, amyrin, and other alcohols were elevated by 76.4%, 257.3%, 451.5%, and 56.3%, respectively (Figure 3B). Notably, the stimulatory effect of GA on primary alcohols and amyrin was particularly striking under drought stress, with increases exceeding 2.5-fold and 4.5-fold, respectively, suggesting a specific role of GA in promoting these two wax fractions under water-limited conditions. In comparison, water priming produced only moderate effects on wax composition under both well-watered and drought conditions (Figure 3), indicating that the observed cuticular modifications were primarily attributable to GA itself rather than to the hydration procedure. Collectively, these results demonstrated that GA priming not only enhanced overall wax deposition but also preferentially modulated specific wax classes, with the most significant changes occurring in response to combined GA application and drought stress.

Sorghum leaf cutin mainly consisted of alkanoic acids (ACs), branched alkanoic acids (BAs), cyclopropaneoctanoic acid (CA), and stearic acid (SC) (Figure 4). Under well-watered conditions, GA priming exerted a positive effect on cutin monomer accumulation, with AC, BA, CA, and OA contents increasing by 60.5%, 140.9%, 83.9%, and 36.3%, respectively (Figure 4A). Drought stress alone also promoted cutin deposition, as evidenced by increases of 204.8% for ACs, 51.5% for BAs, 38.2% for CA, and 60.9% for OA, relative to well-watered controls. When GA priming was applied in addition to drought, the cutin monomer contents were further elevated compared with the drought-only treatment, with ACs and CA showing significant increases of 58.2% and 31.4%, respectively (Figure 4B). By contrast, water priming had no detectable effect on any of the measured monomers under well-watered conditions (Figure 4A), whereas under drought, it significantly reduced the AC content by 42.2% (Figure 4B). Notably, ACs and CA emerged as the most consistently GA-responsive monomers, suggesting a preferential regulation of these two cutin components by GA signaling.

Two-way ANOVA further confirmed significant water × GA interactions for most wax and cutin components, with the exception of aldehyde and stearic acid (Table 1), indicating component-specific regulation by GA signaling.

### 2.4. Effect of Gibberellin Priming on the Cuticular Wax and Alkanoic Acid Carbon Chain Distribution of Sorghum

**Figure 5:**
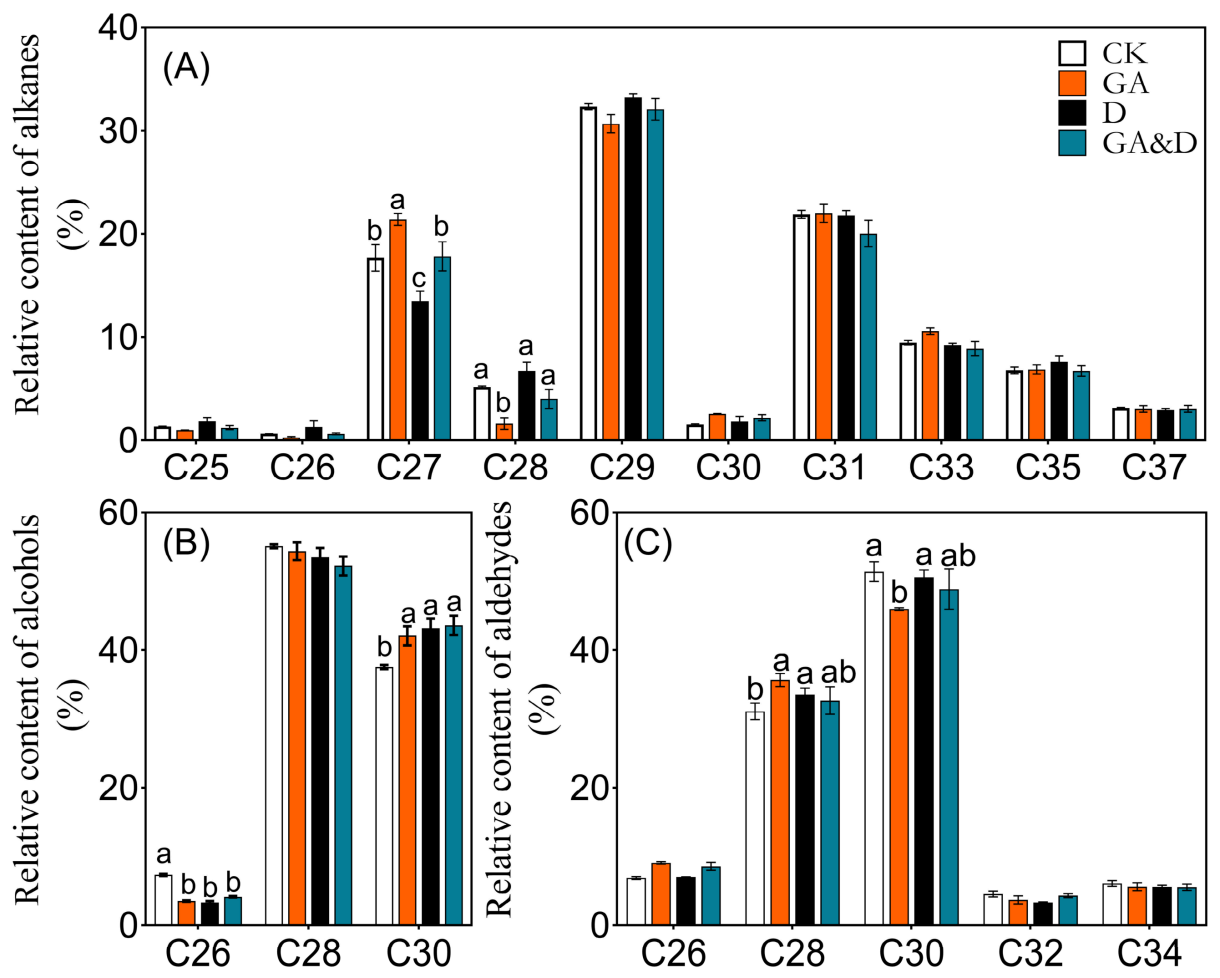
Effects of seed priming with GA on chain length of cuticular waxes: (**A**) n-alkanes, (**B**) n-alcohols, and (**C**) n-aldehydes. The data are presented as the mean ± SE (*n* = 3). CK, plants from non-primed seeds grown under well-watered conditions; D, plants from non-primed seeds grown under drought stress; GA, plants from GA-primed seeds grown under well-watered conditions; GA&D, plants from GA-primed seeds grown under drought stress. Different letters indicate significant differences between bars according to Tukey’s post hoc test (*p* < 0.05); bars sharing the same letter or without letters are not significantly different.

The carbon-chain profiles of the three cuticular wax classes are summarized in Figure 5. Two-way ANOVA showed that GA effects varied by chain length (Table 2). The alkanes predominantly consisted of C25–C37 homologues, with C27 species showing marked responsiveness to GA priming (Figure 5A). GA significantly increased C27 and C28 alkanes, while C33 responded only to drought; none showed water × GA interactions (Table 2 and Table S1). The primary alcohol fraction, which ranges in chain length from C26 to C30 across varieties, exhibited a drought-induced decrease in C26 homologues but a concomitant increase in C30 primary alcohols (Figure 5B). C26 was strongly induced by GA and drought, with a significant interaction (F_1,8_ = 223.95, *p* < 0.001); C30 responded only to water, while C28 was unaffected (Table 2 and Table S1). Similarly, aldehydes (C26–C34) responded to both GA and drought treatments by significantly increasing the accumulation of C28 homologues (Figure 5C). GA reduced C26 independently of water, while C32 showed a significant interaction; C28, C30 and C34 were unaffected (Table 2 and Table S1).

**Figure 6:**
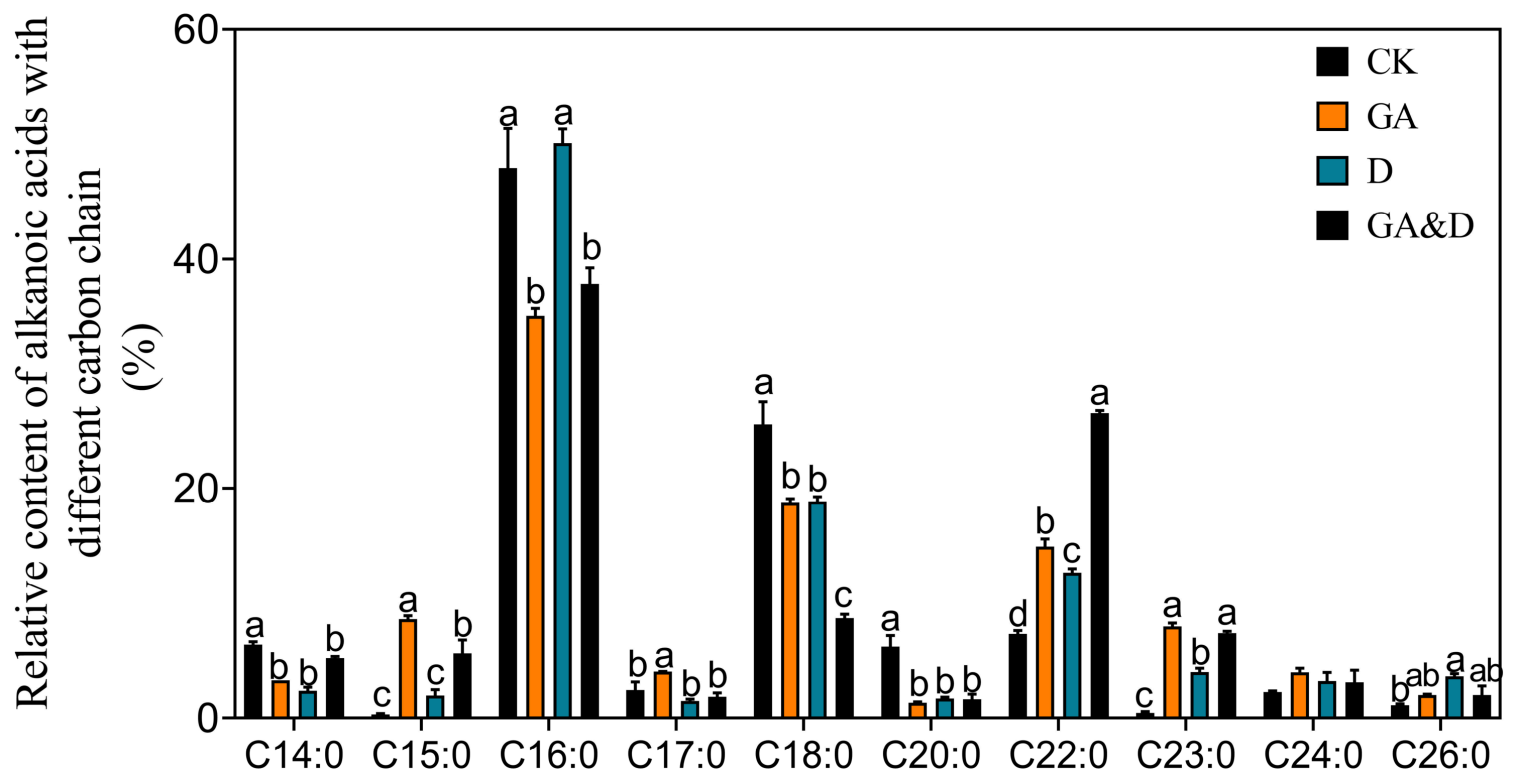
Effects of seed priming with GA on relative abundance of cutin with different chain lengths. Cx:y denotes the number of carbon atoms (x) and double bonds (y) in the alkanoic acid chain. The data are presented as the mean ± SE (*n* = 3). CK, plants from non-primed seeds grown under well-watered conditions; D, plants from non-primed seeds grown under drought stress; GA, plants from GA-primed seeds grown under well-watered conditions; GA&D, plants from GA-primed seeds grown under drought stress. Different letters indicate significant differences between bars according to Tukey’s post hoc test (*p* < 0.05); bars sharing the same letter or without letters are not significantly different.

In contrast, the alkanoic acid pool (C14:0–C26:0) displayed a GA-driven chain-length distribution shift under both well-watered and drought conditions, favoring C22:0 homologues at the expense of C16:0 and C18:0 species (Figure 6). Two-way ANOVA confirmed that GA significantly increased C22:0 under both water regimes (P: F_1,8_ = 633.48, *p* < 0.001), with a significant water × GA interaction (F_1,8_ = 54.28, *p* < 0.001), indicating that this GA effect was further amplified under drought stress (Table 2). GA-mediated increase in C16:0 (P: F_1,8_ = 39.34, *p* < 0.001) occurred independently of water status, as neither the water main effect nor the interaction was significant (Table 2). Responses of other alkanoic acid homologues are summarized in Table S1, with C24:0 being the only homologue that remained completely unaffected by any treatment (*p* > 0.05).

## 3. Discussion

### 3.1. Cuticular Wax Composition and Drought Tolerance

As the first barrier between plants and the external environment, the cuticle’s wax composition (but not its total wax load) is a key determinant of drought tolerance [26]. Plant cuticular waxes are synthesized via two pathways: the alcohol-forming pathway (primary alcohols and wax esters) and the alkane-forming pathway (alkanes, aldehydes, secondary alcohols, and ketones) [27]. In this study, GA priming under drought conditions increased the alkane content by 76.4% and primary alcohol content by 257.3%. The directions of these compositional shifts align with previous observations in other species [28].

Accumulating evidence has established alkanes as critical components of cuticular wax for drought tolerance, and the results from this study provide further support for this function. The drought-induced increase in alkanes following GA priming aligns with observations in tetraploid *Erysimum cheiri* [29] and CsCER1-overexpressing citrus [30], where alkane accumulation contributed to reduced cuticular permeability and enhanced water retention. Moreover, the concurrent reduction in aldehyde content observed in our study warrants attention. As an intermediate in the alkane biosynthetic pathway [31], a decrease in aldehyde content likely reflects accelerated conversion of aldehydes to alkanes rather than impaired biosynthesis. This interpretation is supported by the coordinated alkane increase observed in our study and is consistent with the recently identified CER1-CER3-SOH1 regulatory module that governs substrate partitioning between aldehyde and alcohol formation pathways [31], as well as the reported function of CER1 and CER3 in converting aldehydes to alkanes [32]. Nevertheless, these mechanistic interpretations remain speculative in the absence of direct enzymatic or transcriptional data from our sorghum system. What can be concluded from our data is that GA priming consistently shifted the wax profile toward an alkane-enriched composition, which correlated with reduced cuticular water loss (Figure 2C) and improved physiological performance under drought stress.

### 3.2. Essential Role of Cutin in Cuticular Barrier Function

Cutin forms the structural scaffold of the cuticle, and its integrity is a prerequisite for wax function. Mutants with cutin deficiencies exhibit elevated cuticular permeability even when wax loads remain unchanged [33,34,35,36,37], demonstrating that wax deposition alone cannot compensate for a compromised cutin matrix. This is particularly relevant to this study, as the wax compositional changes would likely translate into improved barrier function only if the cutin scaffold remains intact. The results in this study show that GA priming promoted cutin deposition alongside wax remodeling (Figure 2A,B), supporting the view that the observed reduction in cuticular water loss (Figure 2C) may arise from the combined enhancement of both the scaffold and the wax layer.

GA priming did not induce increases in all cutin monomers; certain components showed preferential accumulation. This pattern is consistent with observations in Arabidopsis [33,38], maize [39,40], and wheat [41], where cutin monomer composition is differentially regulated under drought conditions. The stimulatory effect of GA on cutin deposition was amplified under drought stress, suggesting a synergistic interaction with stress signals. The drought-inducible accumulation of cutin monomers documented across multiple species supports the notion that cutin deposition represents a conserved adaptive response to water deficit [42,43,44,45]. By contrast, water priming exerted negligible or even negative effects on cutin accumulation (Figure 2A,B), confirming the specificity of the effects of GA. Several non-mutually exclusive explanations could account for this inhibitory effect. First, water priming could dilute endogenous GA concentrations or alter the GA-to-ABA ratio through rapid water uptake and subsequent metabolic shifts, thereby attenuating stress-induced cutin biosynthesis [2,3,24]. Second, the transient osmotic shock imposed by water imbibition might trigger a short-term stress response that, under subsequent drought conditions, desensitizes or exhausts the cutin biosynthetic machinery, a phenomenon analogous to the “trade-off” between germination vigor and stress resilience observed in over-primed seeds [46]. These possibilities remain speculative and warrant further investigation through targeted metabolomic and transcriptomic analyses. Nevertheless, the contrasting effects of GA and water priming on cutin deposition underscore the specificity of GA in orchestrating cuticular remodeling under drought stress.

The coordinated accumulation of cutin and wax observed in this study is consistent with improved cuticular barrier function, as reflected by the reduced dehydration rate in GA-primed plants. In rice, the OsWR2 transcription factor simultaneously regulates both cutin and wax accumulation; OsWR2 RNAi mutants exhibit reduced cutin and wax contents accompanied by increased epidermal permeability and reduced drought tolerance, whereas overexpression has the opposite effects [13,47,48]. In barley, drought-induced cutin accumulation is similarly associated with reduced cuticular transpiration and enhanced survival [49,50]. These reports provide a contextual framework for interpreting our observations, although direct causal relationships between specific monomer changes and barrier properties in our sorghum system remain to be established.

Nevertheless, a high cutin content alone does not guarantee effective barrier function. In Arabidopsis, the KCS3 KCS12 double mutant paradoxically accumulates more cutin and wax yet exhibits elevated permeability, indicating that proper assembly and cross-linking of the cutin polyester network are as critical as the total monomer content [51,52,53,54]. The structural organization of cutin, including the polymerization degree and cross-linking density, may therefore be as important as the monomer quantity for barrier functionality.

### 3.3. GA-Mediated Cuticular Lipid Changes

Exogenous GA has been reported to promote cuticle deposition in tomato and apple [55,56], suggesting a conserved role for GA in cuticular lipid metabolism across species. The compositional data in this study showed that GA seed priming in sorghum elicits component-specific rather than uniform cuticular remodeling, with preferential accumulation of alkanes, primary alcohols, and specific cutin monomers (ACs and CA) under drought stress (Figure 3 and Figure 4). The upstream regulatory mechanisms underlying these selective changes were not investigated in the present study and await future exploration. The amplification of the GA effects under drought stress observed in this study raises the possibility of synergistic crosstalk between GA and stress signaling. One possible mechanism involves DELLA proteins, which act as core repressors of GA signaling; their degradation upon detection of GA releases the inhibition of downstream transcription factor activities [57,58]. Drought-induced DELLA accumulation may sensitize the pathway in such a way that GA priming produces a stronger transcriptional response under stress than under well-watered conditions. However, we acknowledge that this DELLA-centered hypothesis is inferred from published work in other species [57,58] and lacks direct experimental support from this study, as we did not measure DELLA protein abundance, GA concentrations, or the transcriptional activity of downstream targets.

### 3.4. Implications for Component-Based Regulation and Drought Tolerance Breeding

In recent years, cuticle-related traits have become important targets for drought tolerance improvement in plants [55,56]. In this study, GA priming induced component-specific changes, alterations to cuticle components in terms of chain length, and cutin–wax coordination. Although the present study provides phenotypic and compositional evidence for GA-mediated cuticle remodeling, the molecular underpinnings—including the transcription factors and biosynthetic genes involved—remain to be elucidated. The component- and chain-length-specific responses in this study provide potential molecular markers for evaluating GA efficacy and could guide the selection of alleles that optimize cuticular barrier properties. However, we emphasize that these implications are preliminary and require validation through targeted genetic and functional studies.

The negative effect of water priming on cutin deposition under drought stress, while beyond the primary scope of this study, represents an intriguing observation that merits future investigation into the metabolic competition between germination-related lipid mobilization and cutin biosynthesis.

While the mechanistic findings of this study were obtained using a single cultivar (Pin05206), the preliminary experiment confirmed that the optimal GA concentration was consistent across all three cultivars tested, suggesting that the underlying physiological principles could have broader relevance. Nevertheless, future studies involving multiple sorghum genotypes under field conditions will be valuable to assess the practical applicability and genotypic robustness of GA priming as a drought management strategy.

## 4. Materials and Methods

### 4.1. Plant Materials, GA Priming, and Drought Stress Treatments

Three sweet sorghum cultivars (Hunnigreen, Mule8000, and Pin05206) were initially used in the preliminary experiment to screen for the optimal GA concentration. As the concentration–response relationship was consistent across all three cultivars, Pin05206—which exhibited the most stable response—was selected for all subsequent mechanistic experiments. Seeds of the hybrid sweet sorghum commercial varieties Hunnigreen and Mule 8000 were provided by Beijing Sanye Grass Seed Lawn Co., Ltd. (Beijing, China). Seeds of Pin05206 were provided by the Institute of Sorghum Research, Shanxi Academy of Agricultural Science, China. The seeds were sterilized with 3% H_2_O_2_ for 5 min, and washed with distilled water to remove any traces of the disinfectant. The seeds were primed with GA (5, 10, 15, 20, and 25 mg L^−1^) at 20 °C in darkness for 24 h [59] before being dried at room temperature until they reached their original weight.

In the first pot experiment, four treatments were used: control (CK), drought stress (D), GA priming under well-watered conditions (GA), and GA priming under drought-stressed conditions (GA&D). CK and GA-primed plants were grown under well-watered conditions (soil moisture content of around 80% of the field capacity). D and GA&D-primed plants at the three-leaf stage were subjected to drought stress for 14 days (soil moisture content of 35% of the field capacity). Water was supplemented every day to keep the soil moisture at the required levels. Four replicates (pots) were established for each treatment, with three seedlings per pot. The experimental soil, with a pH of 6.57, was characterized by 85 mg kg^−1^ alkaline-hydrolyzed nitrogen, 24 mg kg^−1^ Olsen-phosphorus, and 114 mg kg^−1^ exchangeable potassium. A total of 2.5 kg of autoclaved soil was loaded into each container (15 cm in diameter × 20 cm in height). The pots were subsequently arranged in a glasshouse, where the diurnal thermoperiod was regulated at 28 °C (light period) and 20 °C (dark period).

### 4.2. Leaf Cuticle Composition Analysis

Cuticles were extracted from the second fully expanded leaf of drought-treated seedlings after 14 days of treatment. For cutin analysis, 0.03 g of dried, decolorized leaf tissue (bleached in 1:1 CHCl_3_:methanol) was refluxed with 2 mL of methanolic HCl at 80 °C for 2 h. The reaction was quenched with saturated saline, and cutin monomers were extracted with n-hexanes containing hexatriacontane (1 μg) as the internal standard [60]. Surface waxes were collected using three 10 s washes in 10 mL of chloroform containing tetracosane (1 μg). Leaf area was measured prior to wax extraction using a WinFOLIA system (Regent Instrument Inc., Quebec City, QC, Canada) and an EPSON V750 scanner. Both fractions were dried under N_2_ at 40 °C, derivatized with pyridine and BSTFA (20 μL each, 70 °C, 45 min), and the derivatized products were taken up in 1 mL hexane for GC and GC-MS analyses [24,60].

### 4.3. Stress-Related Biochemical and Water Loss

Three oxidative and osmotic stress indicators were analyzed. Leaf proline was extracted using sulfosalicylic acid and colorimetrically assayed according to the method of Bates et al. [61]. The superoxide anion content was spectrophotometrically traced through its oxidation of hydroxylamine according to the method of Elstner and Heupel [62]. Malondialdehyde, a byproduct of peroxidative damage to membrane lipids, was quantified via reaction with thiobarbituric acid, as recommended by Wang et al. [63].

Foliar leaf dehydration under dark conditions was assessed according to the procedure of Burkhardt and Pariyar [64]. In brief, sorghum leaves were dark-acclimated for 12 h to close stomata, and then fully hydrated in distilled water for 1 h to determine the saturated mass. Dehydration was performed in a dark chamber at 25 °C with 70–80% relative humidity, with mass recorded at 15 min intervals for a total duration of 150 min. Dry mass was obtained after 24 h at 70 °C. Water loss was calculated as (saturated mass − fresh mass)/(saturated mass − dry mass) × 100%.

### 4.4. Biomass Analysis

Following the end of the experimental treatment, all tissues were sampled and dried at 70 °C in a forced-air oven for 72 h prior to total shoot dry mass determination.

### 4.5. Statistical Analysis

Values are reported as the mean ± SE of three independent observations (*n* = 3). Data were analyzed using two-way ANOVA with water regime (well-watered vs. drought) and GA priming (control vs. GA-primed) as fixed factors, followed by Tukey’s HSD post hoc test for multiple comparisons. All statistical analyses were performed using IBM SPSS Statistics 17.0 (IBM Corp., Armonk, NY, USA).

## 5. Conclusions

This study demonstrates that GA priming enhances drought tolerance in sorghum through changing the deposition of cutin and cuticular wax, including component-specific wax accumulation, preferential cutin monomer deposition (particularly alkanoic acids and cyclopropaneoctanoic acid), and shifting component species toward longer-chain homologs. These biochemical modifications were associated with reduced water loss, increased proline accumulation, and reduced oxidative damage under drought stress. Our findings establish GA priming as a key regulator of cuticular barrier function and provide a mechanistic basis for employing GA priming to improve crop drought tolerance. Our findings suggest that GA priming represents a promising strategy for enhancing crop drought tolerance through cuticle modification.

## Figures and Tables

**Table 1:** Two-way ANOVA results for cuticular wax and cutin component parameters in sorghum leaves under different water regimes and priming treatments. AC, alkanoic acid; BA, branched alkanoic acid; CA, cyclopropaneoctanoic acid; SC, stearic acid.

Parameter	Water	Primng	W × P
	df = 1	df = 2	df = 2
wax	43.45 ***	92.31 ***	45.41 ***
cutin	193.89 ***	163.06 ***	56.09 ***
alkane	61.14 ***	50.83 ***	21.68 ***
primary alcohol	1.25 ns	94.60 ***	41.58 ***
aldehyde	2.63 ns	7.13 **	2.15 ns
α-amyrin	0.11 ns	75.64 ***	14.51 **
other alcohol	6.71 *	17.17 ***	14.36 **
AC	190.40 ***	129.18 ***	54.49 ***
BA	0.11 ns	4.93 *	5.16 *
CA	1330.14 ***	1376.21 ***	1272.57 ***
SC	0.10 ns	0.76 ns	0.36 ns

Note: ***, *p* < 0.001; **, *p* < 0.01; *, *p* < 0.05; ns, not significant. Two-way ANOVA was performed with water regime (well-watered vs. drought) and priming treatment (control, water-primed, GA-primed) as fixed factors. Error degrees of freedom are 12 for all F-statistics. Values are F-statistics.

**Table 2:** Two-way ANOVA results for carbon chain-length distribution of cuticular wax and cutin components in sorghum leaves under different water regimes and GA priming treatments.

Component	Homologue	Water (W)	GA Priming (P)	W × P
		df = 1	df = 1	df = 1
Alkanes	C27	12.46 **	13.35 **	0.09 ns
	C28	8.09 *	20.55 ***	0.36 ns
	C33	5.69 *	0.91 ns	3.13 ns
Alcohols	C26	119.56 ***	90.60 ***	223.95 ***
	C30	8.58 *	4.06 ns	2.84 ns
Aldehydes	C26	0.32 ns	33.10 ***	0.97 ns
	C32	0.58 ns	0.04 ns	5.83 *
Alkanoic acids	C14:0	22.24 **	0.31 ns	183.13 ***
	C15:0	1.03 ns	84.08 ***	12.52 **
	C16:0	1.52 ns	39.34 ***	0.03 ns
	C18:0	61.81 ***	63.60 ***	2.50 ns
	C20:0	14.62 **	20.40 ***	19.16 **
	C22:0	392.16 ***	633.48 ***	54.28 ***
	C23:0	35.14 ***	473.40 ***	68.03 ***
	C26:0	8.73 *	0.74 ns	8.69 *

Note: ***, *p* < 0.001; **, *p* < 0.01; *, *p* < 0.05; ns, not significant. Two-way ANOVA was performed with water regime (well-watered vs. drought) and GA priming (control vs. GA-primed) as fixed factors. Only homologues with at least one significant effect are shown; non-significant homologues are listed in Table S1. Error degrees of freedom are 8 for all F-statistics. Values are F-statistics.

## Data Availability

All data are contained within the article.

## Supplementary Materials

The following supporting information can be downloaded at: https://www.mdpi.com/article/10.3390/plants15152325/s1, Figure S1: Effects of GA seed priming on sorghum growth and oxidative stress response in sorghum cultivar Hunnigreen. (A) plant height, (B) biomass, (C) proline, (D) total wax, (E) malondialdehyde (MDA) and (F) superoxide anion (O^2−^). The data were means of three replicates ± SE. Different lowercase letters indicate significant differences between treatments according to Tukey’s post hoc test (*p* < 0.05); Figure S2: Effects of GA seed priming on sorghum growth and oxidative stress response in sorghum cultivar Mule8000. (A) plant height, (B) biomass, (C) proline, (D) total wax, (E) malondialdehyde (MDA) and (F) superoxide anion (O^2−^). The data were means of three replicates ± SE. Different lowercase letters indicate significant differences between treatments according to Tukey’s post hoc test (*p* < 0.05); Table S1: Two-way ANOVA results for carbon chain-length distribution of cuticular wax and cutin components in sorghum leaves under different water regimes and GA priming treatments. ***, *p* < 0.001; **, *p* < 0.01; *, *p* < 0.05; ns, not significant. Two-way ANOVA was performed with water regime (well-watered vs. drought) and GA priming (control vs. GA-primed) as fixed factors. Error degrees of freedom are 8 for all F-statistics. Values are F-statistics.

## Abbreviations

The following abbreviations are used in this manuscript:

GA	gibberellin
AC	alkanoic acid
BA	branched alkanoic acid
CA	cyclopropaneoctanoic acid
SC	stearic acid
OA	2.6-oifluorobenzcic acid
