# Supplementary material for: Seed Priming with Gibberellic Acid Enhances Drought Tolerance in Sweet Sorghum by Modulating Cuticular Wax and Cutin Composition
Source: Plants (Basel). 2026 Jul 29;15(15):2325. doi: 10.3390/plants15152325 (PMC13467803; doi:10.3390/plants15152325)
Supplement: Supplementary file 1 [file plants-15-02325-s001.zip › Figure S1.pdf]

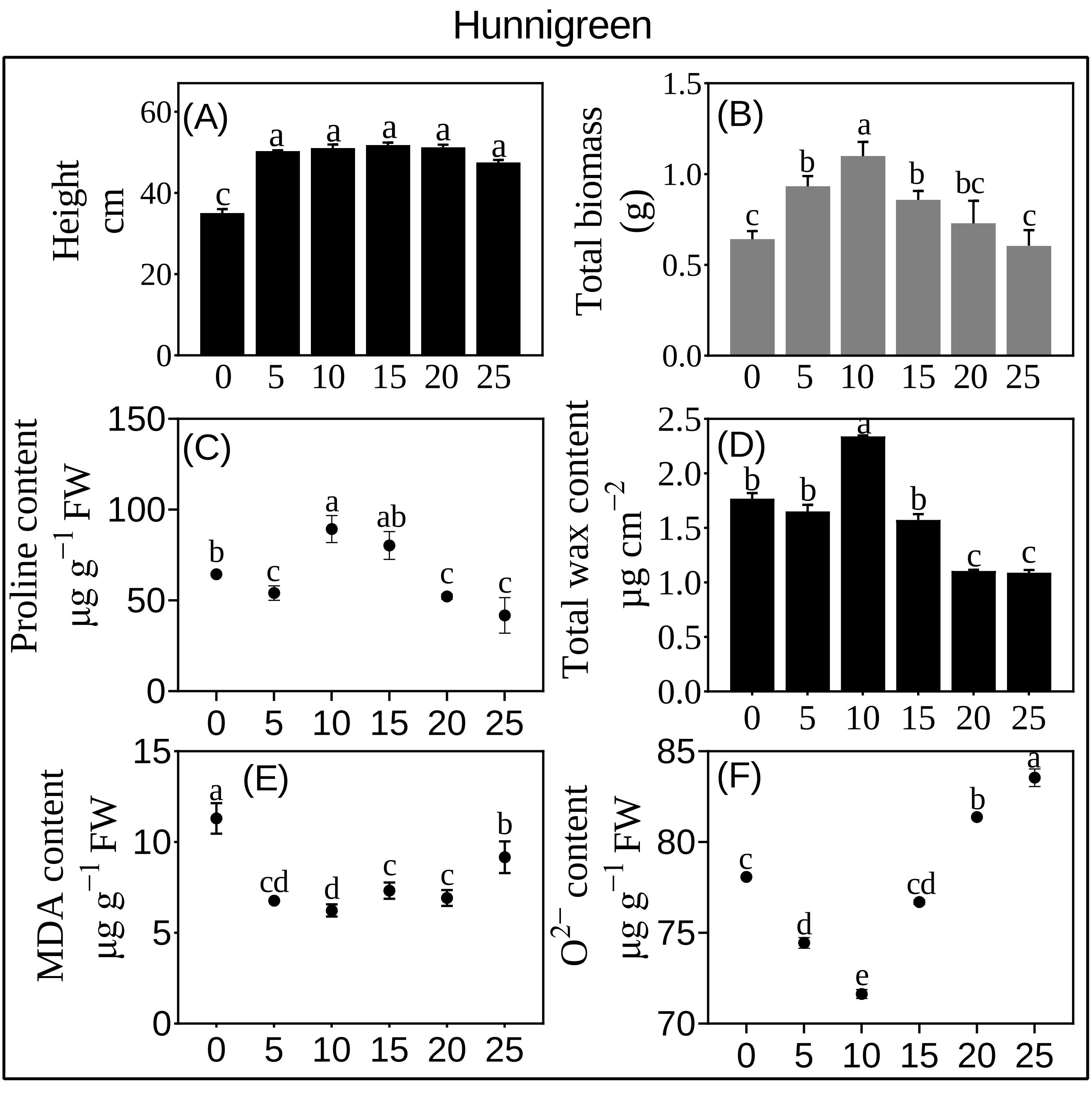

Hunnigreen
(A)
Height cm
(B)
Total biomass (g)
(C)
Proline content µg g^{-1} FW
(D)
Total wax content µg cm^{-2}
(E)
MDA content µg g^{-1} FW
(F)
O^{2-} content µg g^{-1} FW
0 5 10 15 20 25
