# Supplementary material for: Seed Priming with Gibberellic Acid Enhances Drought Tolerance in Sweet Sorghum by Modulating Cuticular Wax and Cutin Composition
Source: Plants (Basel). 2026 Jul 29;15(15):2325. doi: 10.3390/plants15152325 (PMC13467803; doi:10.3390/plants15152325)
Supplement: Supplementary file 1 [file plants-15-02325-s001.zip › Figure S2.pdf]

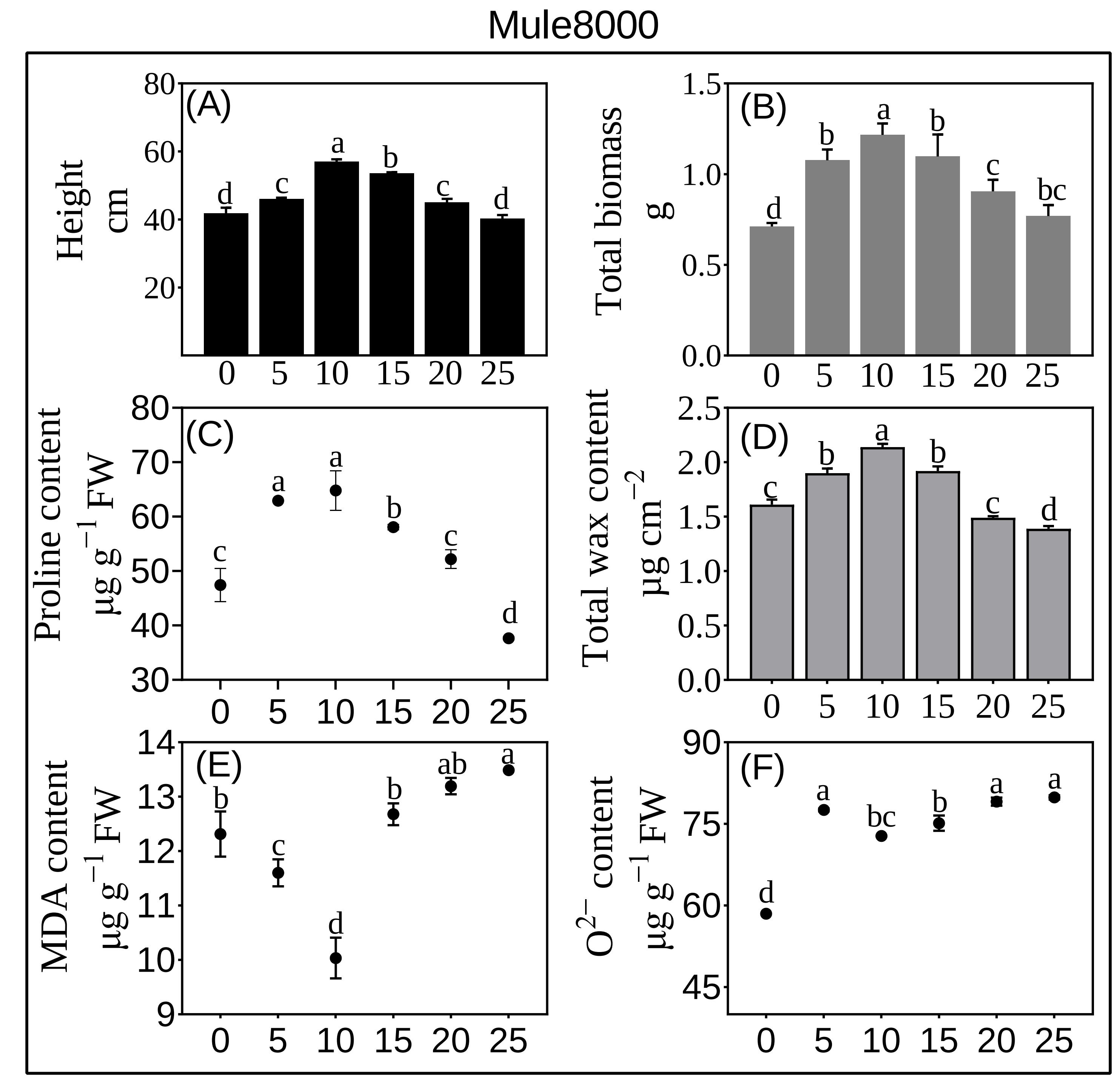
Mule8000
(A)
Height cm
80
60
40
20
0
5
10
15
20
25
d
c
a
b
c
d
(B)
Total biomass g
1.5
1.0
0.5
0.0
d
b
a
b
c
bc
(C)
Proline content μg g−1 FW
80
70
60
50
40
30
c
a
a
b
c
d
(D)
Total wax content μg cm−2
2.5
2.0
1.5
1.0
0.5
0.0
c
b
a
b
c
d
(E)
MDA content μg g−1 FW
14
13
12
11
10
9
b
c
d
b
ab
a
(F)
O2− content μg g−1 FW
90
75
60
45
d
a
bc
b
a
a
